# Anti-EGFR Therapy in Metastatic Colorectal Cancer: Identifying, Tracking, and Overcoming Resistance

**DOI:** 10.3390/cancers17172804

**Published:** 2025-08-27

**Authors:** Luís Felipe Leite, Mariana Macambira Noronha, Junior Samuel Alonso de Menezes, Lucas Diniz da Conceição, Luiz F. Costa Almeida, Anelise Poluboiarinov Cappellaro, Marcos Belotto, Tiago Biachi de Castria, Renata D’Alpino Peixoto, Thais Baccili Cury Megid

**Affiliations:** 1 Department of Medical Sciences, Universidade Federal Fluminense, Niteroi 24220-140, RJ, Brazil; luis_leite@id.uff.br (L.F.L.); lucas_diniz@id.uff.br (L.D.d.C.); almeidaluiz@id.uff.br (L.F.C.A.); 2 Department of Medical Sciences, Universidade Federal do Ceará, Fortaleza 60020-181, CE, Brazil; mariananoronha@alu.ufc.br; 3 Department of Medical Sciences, Universidade Federal da Bahia, Salvador 40170-110, BA, Brazil; juniormenezes@ufba.br; 4 Department of Medical Sciences, Centro Universitário Maurício de Nassau de Barreiras, Barreiras 47808-180, BA, Brazil; ane.cappellaro@gmail.com; 5 Department of Surgery, Hospital 9 de Julho, São Paulo 01409-002, SP, Brazil; marcbelotto@hotmail.com; 6 Department of Gastrointestinal Oncology, H. Lee Moffitt Cancer Center and Research Institute, Tampa, FL 33612, USA; tiago.biachi@moffitt.org; 7 Morsani College of Medicine, University of South Florida, Tampa, FL 33612, USA; 8 Department of Medical Oncology, BC Cancer Agency, Vancouver, BC V5Z 4E6, Canada; renatadalpino@gmail.com; 9 Centre de Formation Médicale du Nouveau-Brunswick, Université de Sherbrooke, Moncton, NB E1C 2Z3, Canada

**Keywords:** metastatic colorectal cancer, EGFR inhibition, negative hyperselection, circulating tumor DNA, precision oncology

## Abstract

Epidermal growth factor receptor (EGFR) inhibitors remain a cornerstone in the treatment of metastatic colorectal cancer with *RAS* and *BRAF* wild-type cancer. Yet, primary and acquired resistance limit their benefit for many patients. A growing body of evidence reveals that resistance is not random but rather driven by a complex network of molecular alterations that sustain tumor growth independent of EGFR signaling. These include amplification of *ERBB2* (HER2) and *MET*, activation of the PI3K and AKT pathways, EGFR extracellular domain mutations, and rare kinase fusions. The concept of negative hyperselection has emerged as a powerful strategy to refine patient selection by excluding tumors with these resistance drivers. Multiple clinical trials have consistently shown that patients who are hyperselected based on comprehensive molecular profiling achieve significantly higher response rates and improved survival compared to those selected by *RAS* and *BRAF* status alone. Liquid biopsy through circulating tumor DNA has further transformed this landscape, offering a noninvasive tool to capture tumor heterogeneity, monitor clonal evolution in real time, and guide rechallenge strategies after resistance emerges. Together, negative hyperselection, ctDNA-guided monitoring, and emerging therapeutics define a precision-oncology framework for identifying, tracking, and overcoming resistance to anti-EGFR therapy in mCRC, moving the field toward more effective and individualized care. Looking ahead, the development of innovative therapeutics such as bispecific antibodies, antibody drug conjugates, and RNA-based therapies promises to further expand in this challenging clinical scenario. These advances move precision oncology in colorectal cancer from concept to clinical reality, reshaping the standard of care through molecular insights.

## Simple Summary

Epidermal growth factor receptor (EGFR) inhibitors are used to treat some patients with metastatic colorectal cancer, but many tumors do not respond or develop resistance during therapy. This review explores how understanding the molecular mechanisms of resistance can help guide treatment decisions. We highlight the concept of negative hyperselection, which uses extended genetic profiling to exclude tumors unlikely to benefit from *EGFR* inhibitors. In addition, we discuss the role of circulating tumor DNA, a blood-based test that monitors tumor evolution in real time. Together, these strategies form a precision oncology platform that supports more personalized and effective care. While these advances offer promise, challenges remain regarding cost, access, and demonstrating meaningful survival benefit in clinical practice.

## 1. Introduction

Colorectal cancer (CRC) is a major global health concern. GLOBOCAN 2020 estimates report close to 2,000,000 new cases and more than 900,000 deaths worldwide in a single year [1]. In the United States, the American Cancer Society projects 153,000 new diagnoses and 52,550 for the year 2023, including almost 20,000 cases under the age of fifty [2]. Although overall mortality continues to decline at roughly 2% per year, incidence in adults younger than 55 years has been rising by about one to two percent annually since the mid-nineteen nineties, and late-stage presentation is again increasing after years of progress toward earlier detection [3]. A gradual shift toward left-sided primary tumors has been noted, with rectal cancer now accounting for approximately one-third of new cases in the United States. This trend is both concerning and clinically relevant in the context of this review, as left-sided tumors typically demonstrate greater responsiveness to anti-EGFR therapy [3,4].

These changing epidemiologic patterns mirror a deeper reality: CRC is not a single homogeneous disease but a spectrum of molecularly distinct entities. This molecular heterogeneity has reshaped how metastatic CRC (mCRC) is treated, moving beyond one-size-fits-all approaches toward strategies guided by tumor biology [5,6]. International guidelines from societies such as the American Society of Clinical Oncology (ASCO), European Society of Clinical Oncology (ESMO), and National Comprehensive Cancer Network (NCCN) recommend routine testing for mutations in *KRAS*, *NRAS*, and *BRAF* [7,8,9]. Tumors that retain the wild-type presentation of these genes are considered eligible for epidermal growth factor receptor (EGFR) blockade with cetuximab or panitumumab, particularly in left-sided tumors [10,11]. Randomized trials demonstrate that the addition of an anti-EGFR antibody to fluoropyrimidine-based doublet or triplet chemotherapy improves response rate and progression-free survival (PFS) compared with chemotherapy alone, and can extend overall survival (OS) in selected patients [12,13].

Identifying the patients most likely to benefit from anti-EGFR antibodies and continuously monitoring the emergence of resistance drivers represents one important frontier in mCRC research and care. This approach allows for the selection of patients who are more likely to respond, while avoiding the administration of ineffective and potentially toxic therapy to those unlikely to benefit. A considerable fraction of patients with *KRAS*, *NRAS*, and *BRAF* wild-type tumors derive little or no advantage from anti-EGFR therapy, and acquired resistance commonly emerges within months of treatment initiation [14,15]. Multiple biological mechanisms account for this variability [15]. The growing recognition of resistance mechanisms has led to the development of more refined strategies to optimize patient selection.

Negative hyperselection is an emerging approach designed to refine patient selection for anti-EGFR therapy in mCRC. It expands molecular profiling beyond the standard evaluation of *RAS* and *BRAF*, aiming to identify additional biomarkers of primary resistance, such as *ERBB2* amplification, *MET* amplification, and rare oncogenic fusions before treatment begins [16]. By excluding tumors harboring these alterations, negative hyperselection seeks to enrich the treatment population for tumors with true EGFR dependency, improving the precision of therapeutic decisions. In parallel, real-time analysis of circulating tumor DNA offers a complementary strategy to monitor the emergence of resistance during therapy, supporting decisions on rechallenge and informing treatment adaptation over time [17].

This review examines how molecular profiling has established a precision oncology platform for EGFR-targeted therapy in mCRC, with a particular focus on the evolving paradigm of negative hyperselection. We discuss the biological basis of primary and acquired resistance, explore how ctDNA complements tissue-based testing, and reflect on how these advances are shaping the broader landscape of mCRC care.

## 2. The EGFR Pathway in Colorectal Cancer

The EGFR plays a central role in regulating cell growth, survival, and proliferation in mCRC. EGFR is a single-pass membrane glycoprotein that couples extracellular growth factor cues to intracellular programmers of proliferation and survival [18]. In its inactive state, the extracellular domain adopts a folded conformation that holds the receptor in a monomeric and catalytically silent configuration [19,20].

Activation begins when ligands such as epidermal growth factor, transforming growth factor alpha, amphiregulin, or epiregulin bind to the extracellular domain. This binding triggers an untethering of the receptor structure, enabling it to form dimers, either with another EGFR molecule or with a partner in the *ERBB* family, such as *HER2*. Dimerization brings together the intracellular kinase domains into an asymmetric alignment, where one kinase lobe activates the other, leading to autophosphorylation of tyrosine residues on the cytoplasmic tail [21,22].

These phosphorylated tyrosines act as docking sites for a variety of adaptor proteins that initiate multiple downstream signaling cascades [23]. One major route involves the adaptor protein GRB2 and the guanine exchange factor SOS, which together activate *RAS* by facilitating GTP loading. This triggers the *RAF–MEK–ERK* cascade, driving gene transcription necessary for cell cycle progression and proliferation [24]. Concurrently, the *PI3K–AKT* pathway is engaged. The p85 subunit of phosphatidylinositol 3-kinase binds to other phosphorylated tyrosines, leading to the production of phosphatidylinositol trisphosphate and subsequent activation of *AKT*. This arm supports metabolic reprogramming, protein synthesis, and resistance to apoptosis, which are key features of tumor survival [25].

Additional branches of the pathway contribute to tumor behavior. Recruitment of phospholipase C gamma generates diacylglycerol and inositol trisphosphate, elevating intracellular calcium and activating protein kinase C, which is involved in regulating cell motility [26]. Simultaneously, phosphorylation events mediated by SRC family kinases facilitate STAT protein docking. Activated STAT3 dimers then translocate to the nucleus, where they promote the expression of key genes including *MYC*, *BCL-xL*, and vascular endothelial growth factor (*VEGF*), further enhancing proliferation, survival, and angiogenesis [18,27].

These convergent routes provide a powerful engine for malignant transformation when feedback checks are lost. Immunohistochemistry detects EGFR protein in 95% of CRCs and intense staining in nearly 80%, whereas true gene amplification is found in fewer than 10%. Overexpression together with abundant autocrine ligand promotes sustained signaling that correlates with poor differentiation, early relapse, and reduced survival [28]. However, despite its frequent expression, EGFR is not directly tested to guide anti-EGFR therapy, as expression levels do not predict response. Instead, the absence of mutations in *KRAS*, *NRAS*, and *BRAF* and other mechanisms determine tumor dependency on EGFR signaling.

Activating mutations in *KRAS* or *NRAS* bypass the need for EGFR signaling by locking the *RAF*, *MEK ERK* pathway in a permanently active state. Similarly, the *BRAFV600E* mutation acts further downstream to provide a continuous growth signal independent of receptor activation [29,30]. In parallel, alterations in *PIK3CA* or loss of *PTEN* hyperactivate the *AKT* pathway, promoting survival and resistance to apoptosis [31,32]. These molecular alterations not only drive tumor progression but also predict poor response to EGFR-targeted therapies, significantly influencing both prognosis and treatment outcomes [33,34].

### Targeting the EGFR Pathway

To understand how a precision oncology paradigm for EGFR inhibitors has emerged, we must first revisit the mechanism of action of these drugs. Recognizing that EGFR’s extracellular domain is both accessible and central to this cascade provided the rationale for developing high-affinity monoclonal antibodies that occupy the ligand pocket, prevent dimerization, and promote receptor down-regulation [35]. Cetuximab is a chimeric immunoglobulin G-one molecule; panitumumab is a fully human immunoglobulin G-two molecule with lower intrinsic immunogenicity [36,37]. Both bind domain III and mediate their antitumor effect through three complementary mechanisms: blockade of ligand-induced signaling, lysosomal degradation of cell-surface receptors, and engagement of immune effector cells through the Fc region (antibody-dependent cellular cytotoxicity is more pronounced with cetuximab because of its IgG-one backbone) [36,38].

In the past decades, early single-arm studies demonstrated activity in chemotherapy-refractory disease, paving the way for randomized evaluations [39,40]. The BOND trial showed that adding cetuximab to irinotecan after prior failure doubled objective response to 23% and tripled PFS to 4.1 months [41]. In the first-line CRYSTAL study, cetuximab combined with FOLFIRI raised response from 40% to 59% and extended PFS from 8 to 10 months among tumors harboring wild-type *KRAS* [42]. The PRIME study established a similar benefit for panitumumab plus FOLFOX, where PFS improved from 7.4 to 9.6 months and overall survival from 19.7 to 23.9 months in extended *RAS*-wild-type disease [43]. Direct comparison in the ASPECCT trial confirmed similar outcomes for these drugs, with OS of 10.4 months for panitumumab and 10 months for cetuximab, and fewer severe infusion reactions with the fully human antibody [44].

However, it was noted that the clinical benefit was strictly contingent on molecular context [45]. Analyses of every pivotal study showed no advantage in tumors carrying activating mutations in *KRAS* or *NRAS* exons 2, 3, or 4, or *BRAFV600E* [46,47]. In left-sided primaries that retain wild-type *RAS* and *BRAF*, cetuximab or panitumumab combined with doublet chemotherapy achieves median OS beyond 35 months, surpassing outcomes obtained with bevacizumab-containing regimens [48]. In right-sided disease, the same antibodies failed to outperform the vascular–endothelial–growth-factor blockade, even when molecularly selected, an observation ascribed to differential embryologic origin, microbiome composition, and cytokine milieu [49,50].

Nowadays, for patients with left-sided, *RAS*-wild-type, and *BRAF*-wild-type metastatic disease, doublet chemotherapy combined with cetuximab or panitumumab is a category-one option. Right-sided disease or tumors harboring *RAS* mutations are better served by chemotherapy plus bevacizumab or, when microsatellite instability is present, immune checkpoint blockade. Tumors with *BRAF V600E* mutations and without microsatellite instability should be treated with FOLFOX, encorafenib, and cetuximab according to the recently published BREAKWATER trial [51]. Additionally, antibody–drug conjugates (ADCs) that link EGFR recognition to cytotoxic payloads, bispecific antibodies that co-target EGFR and *c-MET* or EGFR and HER3, and small interfering RNA nanocomplexes that selectively silence mutant *RAS* are entering early-phase trials, while their results are awaited [52,53,54,55,56,57].

## 3. Mechanisms of Primary Resistance to Anti-EGFR Therapy

**Figure 1:**
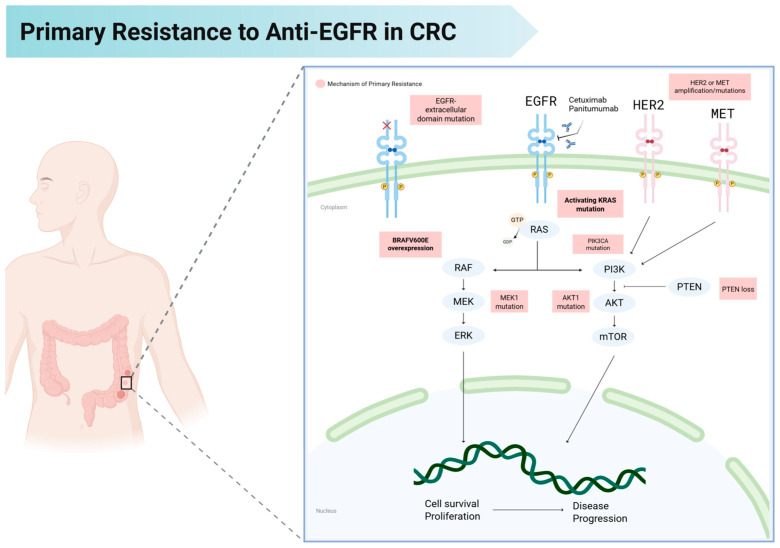
Mechanisms of primary resistance to anti-EGFR therapy in metastatic colorectal cancer. Several genetic alterations drive intrinsic resistance to EGFR-targeted monoclonal antibodies such as cetuximab and panitumumab. These include activating mutations in KRAS and BRAF, amplification or mutation of HER2 and MET, extracellular domain mutations in EGFR, and alterations in downstream signaling components such as PIK3CA, AKT1, MEK1, and PTEN. These molecular events activate parallel or downstream signaling cascades, including the RAS RAF MEK ERK and PI3K AKT mTOR pathways, which sustain tumor cell survival, proliferation, and disease progression independent of EGFR signaling blockade.

Following the demonstration of clinical efficacy with *EGFR*-targeted agents in selected mCRC patients, efforts were made toward mapping the mechanisms of intrinsic resistance to Cetuximab and Panitumumab. Primary resistance is the failure to achieve clinical benefit from anti-EGFR therapy by pre-existing tumor features or genetic alterations that activate downstream signaling independent of EGFR stimulation (Figure 1). Primary resistance is often driven by mutations in the *RAS* gene family (*KRAS* and *NRAS*). Activating mutations in *KRAS* or *NRAS* occur in approximately 40% of mCRC cases, with strong concordance between primary tumors and distant metastases [18,58]. Activating mutations at *KRAS* codons 12 and 13 (exon 2), as well as in *KRAS* exons 3–4 and *NRAS* exons 2–4, impair GTP hydrolysis and lock RAS in its active, GTP-bound form [59]. This constitutive activation drives continuous signaling through the RAF–MEK–ERK (MAPK) pathway, bypassing any blockade of EGFR at the cell surface. Consequently, even if cetuximab or panitumumab successfully inhibits EGFR, the MAPK pathway remains fully active and continues to drive CRC tumor proliferation [60]. Currently, an ongoing phase 3 first-line trial (NCT06252649, CodeBreaK 301) is evaluating whether the addition of anti-KRAS targeted therapy (Sotorasib) to the first line setting is a possible strategy to overcome resistance (Table 1) [61]

Similarly, the *BRAFV600E* mutation bypasses EGFR inhibition by locking the RAF–MEK–ERK cascade in an active state, marking a constitutive resistance. A meta-analysis of 10 randomized control trial demonstrated that neither cetuximab nor panitumumab, alone or combined with chemotherapy, improved OS (HR, 0.91; 95% CI, 0.62–1.34; *p* = 0.63), PFS (HR, 0.88; 95% confidence interval (CI), 0.67–1.14; *p* = 0.33) or (relative risk, 1.31; 95% CI 0.83–2.08, *p* = 0.25) in *BRAF*-mutant mCRC compared with chemotherapy ± bevacizumab [62]. In a retrospective subgroup of the FIRE-3 trial, patients with *BRAF*-mutant tumors achieved almost identical median OS and PFS whether treated with FOLFIRI plus cetuximab or FOLFIRI plus bevacizumab [63]. The phase 1 FIRE-4.5 study then directly compared first-line FOLFOXIRI plus bevacizumab against FOLFOXIRI plus cetuximab in BRAFV600E-mutant mCRC, finding significantly longer PFS (6.7 months v 10.7 months; HR, 1.89; 95% CI, 1.19 to 3.01, 1.89; *p* = 0.006) with bevacizumab despite similar response rates [64]. In recognition of this clinical significance difference, NCCN and ESMO guidelines now recommend testing for *KRAS*, *NRAS*, and *BRAFV600E* mutations to guide the selection of first-line and subsequent therapies [9,65].

### 3.1. HER2 (ERBB2) Amplification and Mutations

*HER2* amplification is a well-established mechanism of resistance to anti-EGFR therapy, occurring in approximately 5–7% of *RAS/BRAF* wild-type mCRC cases [66]. HER2 overexpression actiular sequencing, such as NGSvates downstream signaling, independently of EGFR, bypassed the necessity for EGFR signaling and thus rendered EGFR inhibitors ineffective [67,68]. This pathway redundancy resistance strategy occurs when an amplification of a parallel receptor compensates for the inhibition of EGFR [69]. Clinically, this resistance manifests as a marked reduction in the efficacy of anti-EGFR agents among patients harboring *HER2* amplification, correlating inferior response compared to HER2-negative counterparts. A recent meta-analysis of five retrospective cohort studies involving 594 patients with *RAS* wild-type mCRC demonstrated that HER2-positive tumors treated with anti-EGFR agents exhibited significantly worse PFS (HR 2.84; 95% CI, 1.44–5.60) and ORR (HR 1.96; 95% CI 1.10–3.48) [70].

In response to this clinical challenge, recent trials have explored targeted therapies against HER2 in mCRC. These include antibody-drug conjugates such as trastuzumab deruxtecan (T-DXd), evaluated in the DESTINY-CRC01 trial, which demonstrated an overall response rate of 30.2% and a median PFS of 5.6 months in patients with HER2-positive mCRC refractory to standard therapies, including anti-EGFR agents [71]. Additionally, the HERACLES trial evaluated trastuzumab plus lapatinib, the MyPathway study investigated trastuzumab plus pertuzumab, and the MOUNTAINEER trial assessed trastuzumab plus tucatinib, all demonstrating meaningful responses in heavily pretreated HER2-positive mCRC [72,73,74]. Furthermore, the phase II TRIUMPH (EPOC1602) trial evaluated pertuzumab plus trastuzumab in *RAS* wild-type, *HER2*-amplified mCRC identified by tissue or ctDNA and met its primary endpoint, with confirmed ORRs of 30% (95% CI, 14–50) in the tissue-positive cohort and 28% (12–49) in the ctDNA-positive cohort; median PFS/OS were 4.0/10.1 and 3.1/8.8 months, respectively [75,76].

Collectively, these studies support the clinical validity of HER2 as a therapeutic target in CRC and reinforce the critical importance of routine HER2 testing to guide treatment decisions. HER2-directed therapies are rapidly becoming a new standard of care for this molecularly defined subset [77].

### 3.2. PI3K Pathway Alterations (PIK3CA Mutations, PTEN Loss)

*PIK3CA* mutations and *PTEN* loss activate the PI3K/AKT pathway, promoting tumor survival through mechanisms independent of EGFR [78,79]. These alterations lead to the activation of alternative survival pathways that undermine the efficacy of EGFR-targeted therapies. They are found in approximately 10–20% of CRC, with hotspots in exon 9 and exon 20 [80]. Additionally, loss of function of the tumor suppressor *PTEN*, which normally antagonizes *PI3K* signaling, occurs in about 30% of sporadic CRCs and similarly predicts a lack of response to anti-EGFR treatment [81,82].

The prognostic role of *PIK3CA* mutations were evaluated in a recent meta-analysis of four randomized controlled trials [83]. *PIK3CA* mutations showed no prognostic significance in mCRC. In the control arms without anti-EGFR therapy, OS (HR = 1.11; 95% CI, 0.80–1.55; *p* = 0.54) and PFS (HR = 1.10; 95% CI, 0.72–1.68; *p* = 0.66) did not differ significantly between *PIK3CA* mutant and wild-type tumors. These findings indicate that *PIK3CA* mutations do not impact the natural history of the disease, independent of treatment.

In contrast, the predictive value of *PIK3CA* mutations emerged in the context of anti-EGFR therapy. Patients harboring wild-type *PIK3CA* tumors experienced a significant improvement in PFS with anti-*EGFR* antibodies (HR = 0.57; 95% CI, 0.38–0.87; *p* < 0.01). Conversely, patients with *PIK3CA* mutant tumors derived no significant PFS benefit from anti-EGFR treatment (HR = 0.70; 95% CI, 0.26–1.88; *p* = 0.48). No significant OS benefit was observed in either wild-type (HR = 0.81; *p* = 0.29) or mutant (HR = 0.87; *p* = 0.62) subgroups. These data indicate that *PIK3CA* mutations may modulate sensitivity to anti-EGFR therapy by affecting PFS. Therefore, while *PIK3CA* mutations do not alter overall prognosis, they may serve as biomarkers of resistance to anti-EGFR agents.

### 3.3. MET Amplification

Amplification of the *MET* proto-oncogene, encoding another receptor tyrosine kinase, represents an additional mechanism of bypass signaling [84]. This amplification functions as a parallel signaling route that effectively bypasses the blockade of *EGFR*, sustaining tumor growth despite anti-EGFR therapy. Interestingly, the traditional expansion of ‘MET’ as ‘mesenchymal–epithelial transition factor’ is a common misnomer [85]; the official HGNC designation is ‘MET proto-oncogene,’ and the original ‘met’ acronym was a neutral label from the MNNG-HOS transforming gene [86].

Similarly to *HER2*, *MET* overexpression can activate downstream pathways independently of EGFR, leading to resistance. *MET* activation triggers essential signaling pathways, such as RAS-ERK-MAPK, PI3K-AKT-mTOR, Wnt/β-catenin, and STAT, that drive cellular survival and proliferation and has been proposed as a mechanism to bypass the EGFR-blockade [84]. Therefore, *MET* amplification is included as a resistance marker in hyperselection panels like PRESSING [87].

*MET* amplification is rare, occurring in 1–2% of CRC tumors [14,88]. However, in patients resistant to anti-EGFR therapy, this mutation prevalence is significantly higher, reaching 22.6% (95% CI: 13.31–35.67%) [89]. A phase II study tested tivantinib plus cetuximab in 41 patients with anti-EGFR resistance and *MET* overexpression. The initial phase showed a disease control rate (DCR) of 52.4%, but the trial missed its primary endpoint in the second phase, with only four patients achieving an objective response versus the expected five. Despite this, survival outcomes were encouraging, with a median PFS of 2.6 months and a median OS of 9.2 months [90].

### 3.4. MAP2K1 (MEK1) Mutations

*MAP2K1* is the gene that encodes the protein *MEK1*, a serine/threonine protein kinase that acts downstream of *RAS* in the RAS-RAF-MEK-ERK pathway [91]. These mutations drive resistance to anti-EGFR therapies by constitutively activating the *MAPK* pathway independent of EGFR-inhibition [92]. A recent real-world study screened 246 mCRC patients and found that 2 out of 112 (1.8%) MSS, *RAS/BRAF* wild-type patients harbored the *MAP2K1 K57N* mutation. Both patients received panitumumab monotherapy as third-line treatment and showed primary resistance, with clinical benefit observed only when anti-EGFR agents were combined with cytotoxic chemotherapy [93]. Additionally, although clinical trials have shown the efficacy of combining anti-MEK and anti-EGFR therapies in mCRC with MEK-driven resistance, data specifically addressing resistance in patients with *MAP2K1* mutations remain scarce [94].

### 3.5. AKT1 Mutations

*AKT1* is a serine/threonine kinase involved in the PI3K-AKT-mTOR pathway. Activating mutations in *AKT1* are rare in mCRC, found in less than 2% of cases [95]. These mutations enable alternative signaling pathways that bypass *EGFR* dependency, contributing to resistance against anti-EGFR treatments [96]. *AKT1* mutations frequently co-occur with other genomic alterations within the *PI3K-AKT* pathway or gene amplifications, further complicating therapeutic strategies. A retrospective cohort study of the clinicopathologic and molecular profile to analysis of *AKT1 E17K*-mutated CRC revealed a strong association with mucinous histology (*p* = 0.0006) and frequent co-occurrence with *BRAF V600E* and *KRAS* mutations (*p* = 0.0001) [97]. In this study, 83.3% of *AKT1*-mutated cases harbored concurrent *RAS/RAF* alterations, and two patients with wild-type *RAS* and *BRAF* demonstrated primary resistance to cetuximab, suggesting that *AKT1* mutations may independently contribute to anti-EGFR resistance.

Patients harboring *AKT1* activating mutations often exhibit inferior response outcomes [98]. Many *AKT* inhibitors, AZD5363, GDC-0068, and MK2006, have been tested in early clinical trials [99,100,101]. Investigation of these drugs has focused on *PTEN*-deficient cancers as well as *AKT1*- and *PIK3CA*-mutant tumors.

### 3.6. Gene Fusions

Gene fusions are structural genomic alterations where segments from two different genes become abnormally joined, typically resulting in the expression of a hybrid oncogenic protein. *NTRK* and *RET* fusions are distinct gene rearrangements involving receptor tyrosine kinases that lead to constitutive activation of downstream signaling pathways in mCRC [102,103]. *NTRK* fusions involve the *TRK* family (*NTRK1–3*) and are pan-tumor biomarkers effectively targeted by TRK-inhibitors such as larotrectinib and entrectinib [104]. For example, larotrectinib has shown tumor-agnostic efficacy in a pooled analysis of three trials (adult phase 1, pediatric phase 1–2, adult/adolescent phase 2), with an overall response rate of 75% (95% CI, 61–85) by independent review; at 1 year, 71% of responses were ongoing and 55% of patients remained progression-free, with no discontinuations due to drug-related adverse events [103].

*RET* fusions involve the *RET* (rearranged during transfection) kinase and are targeted by selective RET-inhibitors, including selpercatinib and pralsetinib [105]. Both gene rearrangements drive ligand-independent activation of the *MAPK* and *PI3K-AKT* signaling pathways, bypassing EGFR-inhibition and contributing to resistance to anti-EGFR therapies [106].

Supporting this idea, a large circulating tumor DNA (ctDNA) analysis of 3808 patients with advanced CRC detected gene fusions in 1.1% of cases, most commonly involving *RET*, *FGFR3*, and *ALK* [107]. These fusions were frequently subclonal and often co-occurred with *RAS* and *EGFR* mutations associated with anti-EGFR resistance. Notably, 78% of patients with clinical data had received EGFR-targeted therapy before fusion detection, reinforcing the hypothesis that these fusions may emerge as novel mechanisms of acquired resistance to anti-EGFR treatment in mCRC. Overall, identifying targetable gene fusions offers a path forward to expand options in mCRC—allowing a small but meaningful subset of patients to active, tumor-agnostic therapies [108].

**Table 1:** Primary mechanisms of intrinsic resistance to anti-EGFR therapy in metastatic colorectal cancer (mCRC).

Mechanism of Primary Resistance	Prevalence	Clinical Impact in Patients Treated with Anti-EGFR Therapy (Effect Size, 95% CI)	Possible Strategies to Overcome Resistance	References
*KRAS*/*NRAS* activating mutations	40%	OS: HR 1.06 (0.96–1.17)	Addition of anti-KRAS targeted therapy (e.g., Code BreaK 301 trial)	[61,109,110]
*BRAF V600E* mutations	8–12%	OS: HR 0.91 (0.62–1.34); PFS: HR 0.88 (0.67–1.14); ORR: RR 1.31 (0.83–2.08)	Combined EGFR and BRAF inhibitors (e.g., BREAKWATER)	[47,111,112]
*HER2* (*ERBB2*) amplification	5–7%	PFS: HR 2.84 (1.44–5.60); ORR: OR 1.96 (1.10–3.48)	HER2-targeted therapy (e.g., HERACLES-B trial, TRIUMPH trial)	[71,72,75,113]
*PIK3CA* mutations/*PTEN* loss	10–20%	PFS: WT HR 0.57 (0.38–0.87) ^†^; mutant HR 0.70 (0.26–1.88)	Addition of PI3K or mTor inhibitors	[73,81,82,114,115]
*MET* amplification	1–2% baseline; 22% after anti-EGFR	OS: HR 1.33 (1.06–1.59); PFS: HR 1.47 (1.03–1.91) ^‡^	Dual EGFR/MET Inhibition (e.g., Tivantinib + Cetuximab)	[85,90,116,117]
*MAP2K1* (*MEK1*) mutations	1.8% (MSS, RAS/BRAF WT cohort)	Data remains limited to small case series/reports	Combined BRAF + EGFR + MEK1 inhibitiors	[118,119,120]
*AKT1* mutations	<2%	Data remains limited to small case series/reports	AKT inhibitors	[94,121]
Oncogenic fusions (*NTRK*/*RET*/*ALK*/*ROS1*/*FGFR3*)	1%	Data remains limited to small case series/reports	Fusion-targeted therapies (e.g., TRK, RET inhibitors)	[96]

The table summarizes prevalence, clinical impact in anti-EGFR–treated cohorts (reported OS, PFS, ORR effect sizes with 95% CIs), and example strategies to overcome resistance. Abbreviations: OS, overall survival; PFS, progression-free survival; ORR, objective response rate; WT, wild type; NR, not reported; mTOR, mechanistic target of rapamycin; MSS, microsatellite stable. Notes: ^†^ For *PIK3CA*, PFS benefit with anti-EGFR therapy is confined to WT tumors; no significant benefit is seen in PIK3CA-mutant disease. ^‡^ For *MET*, the listed HRs reflect the prognostic impact of high c-MET expression in CRC overall and are not anti-EGFR-specific estimates.

## 4. Mechanisms of Acquired Resistance to Anti-EGFR Therapy

**Figure 2:**
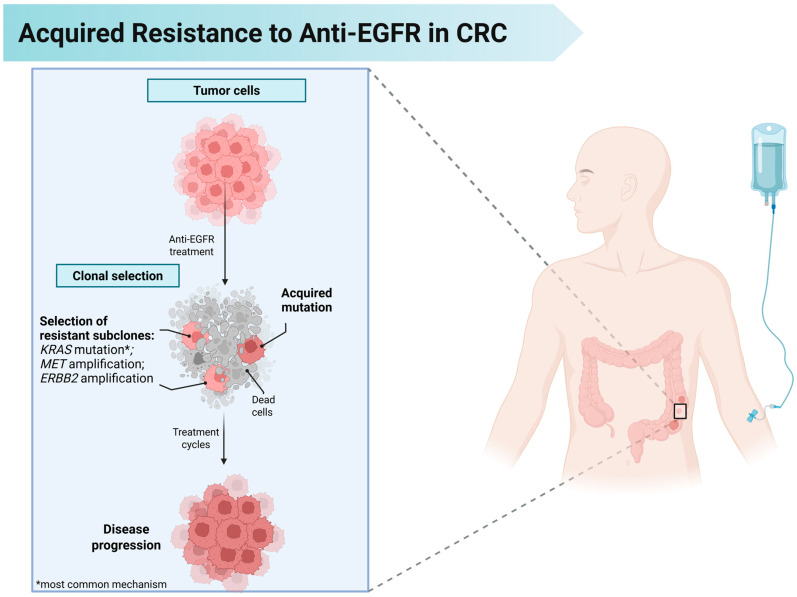
Mechanisms of Acquired Resistance to Anti-EGFR Therapy in Metastatic Colorectal Cancer. EGFR-targeted monoclonal antibodies (cetuximab or panitumumab) initially reduce tumor burden in RAS/BRAF wild-type colorectal cancer. However, under treatment pressure, resistant clones (e.g., with KRAS mutations) are positively selected or newly emerge through acquired mutations. These resistant subclones expand over subsequent treatment cycles, ultimately leading to disease progression despite continued anti-EGFR therapy.

Even among patients initially responsive to anti-EGFR therapy and without primary resistance, disease progression occurs. Intratumoral heterogeneity is a central feature of colorectal cancer and decisively influences this acquired resistance [108]. In fact, CRC tumor consists of a complex population of distinct cellular clones that, over time, accumulate various genetic and epigenetic alterations, including mutations in oncogenes and tumor suppressor genes, DNA methylation changes, and variations in microRNA expression [122,123]. This diversity results from a branched evolutionary process driven by genomic instability, which generates clonal variation, and by the natural selection of more adapted clones, favored by the pressures of the tumor microenvironment and applied therapies (Figure 2).

In the context of *KRAS* wild-type mCRC, we detailed previously that anti-*EGFR* therapy demonstrates significant efficacy [8]. However, intrinsic tumor heterogeneity and genomic instability promote the rapid emergence of resistant subclones, leading to a progressive decline in response and disease progression, with acquired resistance typically manifesting within 12 months. This phenomenon reflects a dynamic tumor ecosystem in which pre-existing or newly emerged subpopulations, endowed with adaptive advantages, expand under the selective pressure imposed by therapy [124]. The identification of the main molecular alterations responsible for this resistance has been a priority for the development of personalized therapeutic strategies, prompting a series of clinical trials aimed at understanding the mechanisms involved in acquired resistance [125,126,127].

Genetic alterations in *KRAS*, such as mutations and amplifications, are present in approximately 50% of cases and represent the main mechanism of acquired resistance in this scenario [128]. These alterations may arise from the expansion of pre-existing mutated clones or from the emergence of new mutations induced by the selective pressure exerted by prolonged use of agents such as cetuximab or panitumumab [129,130]. Moreover, they may coexist with other genomic alterations that promote the activation of alternative signaling pathways, further compromising the efficacy of the anti-EGFR blockade [131]. Although less frequent, secondary mutations also contribute significantly to acquired resistance to anti-*EGFR* therapy [132]. Among these, mutations in the extracellular domain III of *EGFR* are particularly notable, as this region is crucial for cetuximab recognition; such mutations impair antibody binding to the receptor and reduce its therapeutic efficacy [133]. Additionally, mutations in *BRAF*, a member of the *RAF* serine/threonine kinase family, promote continuous activation of the *RAF-ERK* pathway, constituting an important mechanism of acquired resistance [134,135]. *ERBB2* amplifications, which encode the *HER2* receptor, may also be induced by cetuximab treatment, resulting in persistent activation of the *ERK1/2* pathway [136]. In parallel, the selective pressure exerted by anti-*EGFR* therapies favors the expansion of subclones with *MET* amplification, further contributing to acquired resistance [84]. The coexistence of these mechanisms underscores the need for a deeper understanding to develop more effective therapeutic approaches. Beyond genomic alterations, there is evidence that acquired resistance may also be mediated by transcriptomic profiles associated with epithelial–mesenchymal transition, characterizing a pattern of cross-resistance [137]. Additional preclinical data indicate that cancer-associated fibroblasts (CAF), through the secretion of various soluble factors, play a central role in inducing resistance to EGFR blockade [137,138]. Key CAF-derived factors implicated in anti-EGFR resistance include HGF (activates MET and sustains MAPK/PI3K–AKT signaling), TGF-β (promotes EMT and adaptive tolerance), IL-6/IL-8 (STAT3-mediated survival/inflammation), FGF2 (FGFR signaling), and neuregulin-1 (HER3/HER2 activation) [139]. By sustaining parallel pathways despite EGFR blockade, these stromal interactions highlight the importance of considering the tumor microenvironment as a therapeutic target [140,141]. These findings support the notion that therapeutic resistance mechanisms are not limited to genomic alterations in the molecular target but also include transcriptomic adaptations and complex interactions with the tumor microenvironment [60].

## 5. Hyperselection of Patients for Anti-EGFR Therapy

After elucidating the diverse and complex mechanisms underlying resistance to anti-EGFR therapy, it became clear that traditional selection criteria were insufficient to fully identify patients who would benefit from these agents. This realization prompted the emergence of a new precision oncology paradigm: negative hyperselection. Rather than selecting patients based solely on the presence of favorable biomarkers, this approach seeks to exclude tumors harboring known resistance drivers, even when conventional biomarkers suggest eligibility [10]. In other words, even after the traditional selection based on *RAS* and *BRAF* and tumor sidedness, approximately one-third of patients do not respond to *EGFR*-inhibition, indicating that additional molecular escape mechanisms limit therapeutic efficacy [142]. This approach exemplifies how a deep understanding of molecular mechanisms can directly inform clinical decision-making, bridging the gap between genomic insight and patient care.

As detailed in previous sections, alterations such as *HER2* and *MET* amplifications, *NTRK*, *ROS1*, *ALK*, and *RET* fusions, *PIK3CA* exon 20 and *MAP2K1* mutations, as well as *PTEN* loss, activate alternative signaling pathways that circumvent *EGFR*-blockade [143]. Mapping the presence of these alterations before treatment initiation could enhance patient selection.

In this context, the PRESSING case–control study was landmark in expanding the understanding of molecular mechanisms behind resistance to this therapy. It evaluated whether a selected panel of genomic alterations (the PRESSING panel) could predict resistance to anti-*EGFR* antibodies beyond common *RAS* and *BRAF* mutations. The panel included *HER2* and *MET* amplifications, fusions involving *NTRK*, *ROS1*, *ALK*, and *RET*, and *PIK3CA* exon 20 mutations. The key finding was that PRESSING-negative patients (without these alterations) showed significantly greater clinical benefit, with response rates potentially increasing from 50% to 67% and longer PFS (median 6.3 vs. 2.7 months). Although OS was not significantly affected, these alterations appear important in predicting treatment resistance.

Furthermore, tumor sidedness retained its prognostic impact even after molecular hyperselection: right-sided tumors displayed a higher frequency of resistance-associated alterations (41.4% vs. 20%) and poorer response to treatment (PFS of 3.3 vs. 7.3 months). The combination of tumor sidedness with the PRESSING panel increased the predictive accuracy to approximately 80%, underscoring the importance of integrating both anatomical and molecular variables in therapeutic decision-making. The PRESSING panel also demonstrated that microsatellite instability (MSI-high), present exclusively in resistant patients, is strongly associated with these alterations and predominates in right-sided tumors, highlighting the molecular complexity of mCRC.

Building on these promising results, new studies have explored the clinical role of negative hyperselection in managing mCRC. Notably, the randomized phase II PANDA trial evaluated chemotherapy plus anti-EGFR therapy in elderly patients with *RAS/BRAF* wild-type mCRC, stratified by a modified PRESSING panel that included *MAP2K1* mutations and *PTEN* loss expression [16]. Molecular hyperselection was independently linked to significant improvements in PFS (12.8 vs. 7.6 months) and OS (29.5 vs. 20 months), a benefit not seen in the original PRESSING study. These findings confirm the clinical importance of these rare mutations in *EGFR* resistance and support hyperselection as a strong molecular strategy to identify ideal candidates for anti-EGFR therapy in clinical practice.

Exploratory analyses of the randomized phase II PanaMa trial evaluated the role of negative hyperselection in maintenance therapy with panitumumab in patients with *RAS* wild-type mCRC [140]. All participants received induction with mFOLFOX6 combined with panitumumab, followed by randomization to maintenance with chemotherapy alone or in combination with the anti-EGFR agent. The results confirmed the prognostic value of negative hyperselection, evidenced by superior PFS and OS in patients without mutations compared to those with at least one identified alteration (median OS: 28.7 vs. 22.2 months; significant). In a comparative context of maintenance with or without panitumumab, negative hyperselection proved to be an effective predictive biomarker capable of identifying patients with the greatest potential benefit.

Taking a step further, current studies aim to enhance both the precision and the breadth of detectable mutations. The PRESSING2 study employed an expanded molecular panel to identify uncommon genomic alterations in RAS wild-type mCRC patients undergoing anti-EGFR therapy [87]. This panel incorporated potential novel oncogenic alterations relative to the original PRESSING panel, including mutations associated with the *MAPK* pathway, *PIK3CA*, *EGFR*-independent tyrosine kinase signaling routes, and *EGFR* rearrangements affecting the tyrosine kinase domain. PRESSING2-positive patients exhibited significantly worse survival, suggesting that an expanded molecular panel may refine the selection process for anti-EGFR therapy. Table 2 presents the main clinical studies evaluating the role of negative hyperselection.

## 6. The Role of Circulating Tumor DNA in Tracking Resistance to Anti-EGFR Therapy

While negative hyperselection enhances baseline patient stratification, resistance to anti-*EGFR* therapy often emerges dynamically under treatment pressure. This has positioned liquid biopsy and ctDNA as a critical component of the precision oncology platform for mCRC treatment.

Historically, molecular profiling for CRC has relied on analyzing tumor tissue obtained via biopsy or surgical resection (from either the primary tumor or a metastatic site) [85]. Formalin-fixed paraffin-embedded samples remain the reference for detecting oncogenic drivers and copy number alterations, such as *HER2* or *MET* amplification. Yet tissue captures only one spatial and temporal frame of a disease that is intrinsically heterogeneous and continually evolving under therapeutic pressure. Repeated biopsy is often impractical, sometimes impossible, and it cannot reveal the dynamic rise and fall of subclones that ultimately determine clinical outcome [147]. Despite its capabilities, tissue-based next generation sequencing (NGS) has several inherent limitations, such as intratumor heterogeneity, lack of analysis of dynamic changes and clonal evolution that occur over time (particularly in the presence of therapy), and practical execution difficulties due to challenges in obtaining sufficient quantity and quality of tumor tissue, often compounded by tumor location or the patient’s condition [122,148].

ctDNA, small DNA fragments shed by tumors into the bloodstream, allows for non-invasive liquid biopsies for genomic profiling via molecular sequencing, such as NGS [149]. Compared to tissue biopsies, ctDNA analysis offers several advantages: it is non-invasive and repeatable, can better capture tumor heterogeneity by sampling DNA from multiple sites, enables real-time monitoring of tumor evolution and treatment response (including early detection of resistance), serves as a sensitive marker for minimal residual disease (MRD) post-treatment to predict recurrence, and potentially offers faster turnaround times [150,151,152,153].

**Figure 3:**
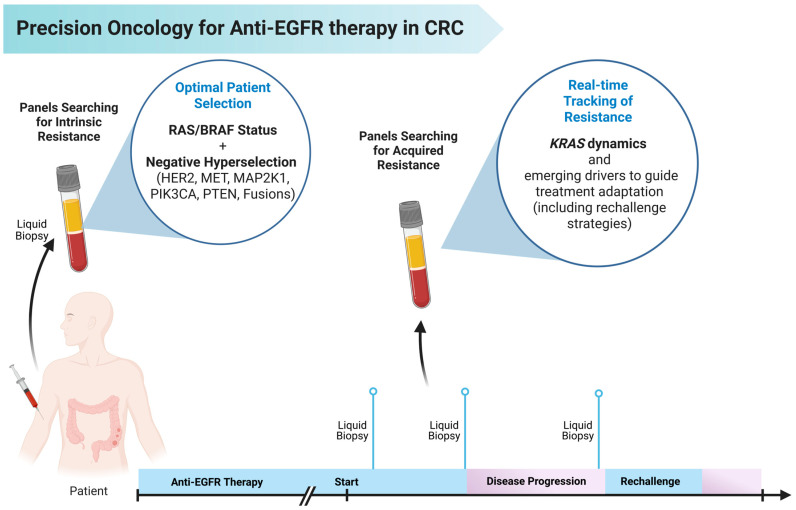
Precision Oncology Framework for Anti-EGFR Therapy in Metastatic Colorectal Cancer. Liquid biopsy enables noninvasive, real-time molecular profiling across the treatment course. At baseline, panels assessing *RAS*/*BRAF* status and additional negative hyperselection markers (e.g., *HER2*, *MET*, *MAP2K1*, *PIK3CA*, *PTEN*, gene fusions) identify patients most likely to benefit from EGFR blockade. During treatment and at disease progression, serial ctDNA analysis detects acquired resistance mechanisms, supporting rechallenge decisions or the addition of targeted agents. This dynamic strategy integrates predictive and adaptive molecular insights to optimize anti-EGFR therapy.

In anti-EGFR therapy for mCRC, ctDNA is valuable for baseline stratification (detecting primary resistance like RAS or *BRAF* when tissue is limited), monitoring response and resistance (detecting changes in mutation frequency and emergence of new resistance mechanisms like *EGFR ECD* mutations), and guiding rechallenge strategies based on the presence or absence of prior resistance mutations [60,154,155] (Figure 3). In a recent meta-analysis including 402 patients retreated with cetuximab or panitumumab after an *EGFR*-free interval, the pooled objective response rate was 20.5% and the disease control rate reached 67.4%. The median PFS and OS were estimated at 3.5 and 9.8 months, respectively. Notably, patients selected for rechallenge based on the absence of *RAS* mutations in ctDNA experienced significantly improved survival, with a pooled hazard ratio for death of 0.41 compared to those with detectable *RAS* mutations. These findings underscore the clinical utility of ctDNA not only as a biomarker but as a real-time decision-making instrument in the management of anti-EGFR resistance [17].

Despite its promise, ctDNA analysis presents several limitations currently. Its sensitivity is affected by tumor biology and assay performance [156,157,158]. False-negative results may occur in cases with low tumor burden, limited tumor shedding, or metastases located in compartments with restricted access to the bloodstream, such as the peritoneum or central nervous system [159,160]. Turnaround time, although generally shorter than that of tissue-based sequencing, may still be suboptimal due to logistical delays in sample handling and reporting [161,162]. Financial and infrastructural barriers also limit access to ctDNA testing in many healthcare settings [163].

Clinically, the interpretation of ctDNA results is not always straightforward. The presence of resistance mutations does not uniformly indicate a lack of benefit, and absence of detectable alterations does not ensure durable response. Outside of validated settings such as *RAS* testing for anti-EGFR therapy or minimal residual disease detection, the impact of ctDNA-guided interventions on overall survival remains to be established [164].

In summary, resistance to anti-*EGFR* therapy, whether intrinsic or acquired, remains a major obstacle in the treatment of metastatic colorectal cancer. This challenge has driven the development of more refined molecular stratification strategies, including the concept of negative hyperselection, which seeks to exclude tumors that harbor additional resistance drivers beyond *RAS* and *BRAF* mutations. In parallel, circulating tumor DNA has emerged as a valuable tool for real-time monitoring of clonal evolution during therapy, capturing the dynamic emergence of acquired resistance and informing decisions such as anti-EGFR rechallenge. Concurrently, emerging modalities, including bispecific antibodies, EGFR-directed antibody–drug conjugates, and RNA-based therapeutics will expand the options for patients with resistant disease [53,158,165,166]. Linking the targetable molecular alterations to matched strategies enables more personalized care. Together, these approaches illustrate how precision oncology is transforming the management of colorectal cancer by replacing static, one-time molecular assessments with a continuous and adaptive model of care.

Selecting patients most likely to benefit from EGFR blockade, directing others to alternative targeted options, and ultimately aiming for improved survival and quality of life while minimizing unnecessary toxicity seems to be the goal in this setting. This paradigm shift must be accompanied by careful consideration of cost effectiveness, demonstrated survival benefit, and equitable access. As the field advances, integrating molecular complexity into routine practice must be balanced with the responsibility to ensure that innovations translate into meaningful improvements in patient outcomes.

## 7. Conclusions

Optimizing anti-EGFR therapy in mCRC demands more than *RAS* and *BRAF* testing. A growing body of evidence shows that resistance arises from a complex network of alterations, including *HER2* and *MET* amplification, *PI3K* pathway activation, and rare oncogenic fusions. The concept of negative hyperselection, which systematically excludes these resistance drivers, has consistently translated into improved response rates and survival across multiple clinical trials. Liquid biopsy has further transformed this landscape. Circulating tumor DNA enables real-time tracking of clonal evolution, early detection of resistance, and more effective rechallenge strategies. This approach offers a dynamic complement to tissue-based profiling and a pragmatic framework for real-world decision-making, with the potential to improve outcomes while reducing ineffective exposure, toxicity, and costs.

The future of EGFR-targeted therapy lies at the intersection of deeper molecular refinement and adaptive treatment strategies. Emerging modalities such as bispecific antibodies, antibody drug conjugates, and RNA-based therapeutics promise to expand treatment options for patients previously considered resistant. Successful implementation of liquid biopsy will require validated and standardized assays, timely turnaround, equitable access, and prospective evidence demonstrating survival benefit and cost-effectiveness. Precision oncology in CRC is no longer aspirational. It is becoming the standard, driven by molecular insight and continuous innovation.

## Figures and Tables

**Table 2:** Clinical Studies evaluating the role of negative hyperselection in mCRC.

Study	Patient Population	Biomarkers Evaluated	Overall Response Rate (%)	Median PFS (Months)	Median OS (Months)	Key Findings
PanaMa Trial [140]	mCRC, RAS WT	*BRAF V600E/PIK3CA/AKT1/ALK/ERBB2/PTEN MUT and HER2/neu*	35.8% in hyperselected vs. 25%	7.5 months in hyperselected vs. 4.4 months	28.7 months in hyperselected vs. 22.2 months	Hyperselected patients had significantly better PFS and OS with panitumumab maintenance.
Valentino Study [144]	mCRC, RAS WT	*HER2/MET PIK3CA/PTEN* mutations	N/A	10.5 months in hyperselected vs. 6.03 months	33.3 months in hyperselected vs. 14.1 months	Reinduction with panitumumab was more effective in hyperselected patients.
PANDA Trial [145]	Elderly mCRC, RAS WT, BRAF WT	*HER2/MET NTRK/ROS1/ALK/RET PIK3CA PTEN AKT1 MAP2K1*	71% in hyperselected vs. 51%	12.8 months in hyperselected vs. 7.6 months	29.5 months in hyperselected vs. 20 months	Elderly hyperselected patients showed better PFS and OS with anti-EGFR therapy.
PRESSING2 Study [87]	mCRC, RAS WT, MSS, POLE WT	*ERBB2/MET NTRKs/RET/ROS1/ALK AKT1/PTEN/PIK3CA*	79% in hyperselected vs. 56%	12.8 months in hyperselected vs. 6.4 months	49.9 months in hyperselected vs. 22.6 months	Ultraselected patients (without rare mutations) had significantly better survival outcomes.
PARADIGM Study [17]	mCRC, RAS WT	*PTEN/EGFR HER2/MET ALK/RET/NTRK1*	N/A	N/A	41.4 months in hyperselected vs. 18.7	Exploratory analyses suggest that ctDNA is useful to identify gene alterations that predict resistance to *EGFR* inhibition.
Morano et al. [146]	mCRC, RAS/BRAF WT	*ALK/ROS1/NTRKs/RET HER2/PIK3CAex.20/PTEN/AKT1*	75.3% in hyperselected vs. 59.2%	12.1 months in hyperselected vs. 7.7	68.1 months in hyperselected vs. 48.1	Patients with right-sided tumors and those without hyperselected tumors had significantly poor outcomes in terms of overall ORR, PFS, and OS.

*BRAF WT*: *BRAF* Wild Type; ctDNA: Circulating Tumor DNA; FOLFOX: (Oxaliplatin, Leucovorin, and 5-Fluorouracil); mFOLFOX6: Modified FOLFOX6 (Oxaliplatin, Leucovorin, and 5-Fluorouracil); mCRC: Metastatic Colorectal Cancer; Median OS: Median Overall Survival; Median PFS: Median Progression-Free Survival; MSS: Microsatellite Stable; 5-FU/FA: 5-Fluorouracil/Folinic Acid; *POLE WT*: *POLE* Wild Type; *RAS WT*: *RAS* Wild Type; N/A: Not available.

## Data Availability

The data underlying this study are extracted from publicly available studies.
